# Behavioral Effects of Renin-Angiotensin System Modulators in a Rotenone-Induced Model of Parkinson’s Disease in Wistar Albino Rats

**DOI:** 10.7759/cureus.109052

**Published:** 2026-05-17

**Authors:** Prakash KG, Saniya K, Aishwarya Mantri, Madhavrao Chavan, Mythili Bai K, Shubham Jaju

**Affiliations:** 1 Anatomy, Pacific Institute of Medical Sciences, Sai Tirupati University, Udaipur, IND; 2 Pathology, Pacific Institute of Medical Sciences, Sai Tirupati University, Udaipur, IND; 3 Pharmacology, All India Institute of Medical Sciences, Mangalagiri, Mangalagiri, IND; 4 Physiology, All India Institute of Medical Sciences, Mangalagiri, Mangalagiri, IND; 5 Pharmacology, Pacific Institute of Medical Sciences, Sai Tirupati University, Udaipur, IND

**Keywords:** neuroprotection, parkinson’s disease, renin–angiotensin system, rotenone, wistar rats

## Abstract

Introduction: Parkinson’s disease (PD) is a progressive neurodegenerative disorder characterized by motor and non-motor dysfunction resulting from dopaminergic neuronal loss. Emerging evidence suggests a role of the renin-angiotensin system (RAS) in neurodegeneration, making it a potential therapeutic target. The present study aimed to evaluate the behavioral effects of captopril, perindopril, and losartan on motor, behavioral, and anxiety parameters in a rotenone-induced model of Parkinson’s disease in Wistar albino rats.

Materials and methods: An experimental in vivo study was conducted using a rotenone-induced PD model in Wistar albino rats (n = 6 per group). Animals were divided into six groups: vehicle control, rotenone control, standard treatment group (levodopa + benserazide), and treatment groups receiving captopril, perindopril, or losartan along with rotenone. Neurobehavioral assessments included locomotor activity, rotarod performance, wire-hanging test, hole board test, forced swim test, and elevated plus maze. Data were analyzed using one-way analysis of variance (ANOVA) followed by Tukey’s post hoc test.

Results: Rotenone administration significantly impaired motor and behavioral parameters, with reduced locomotor activity (18.67 ± 4.11 vs 174.33 ± 25.01 counts/10 min), rotarod performance (10.17 ± 1.19 vs 118.17 ± 1.83 sec), and wire-hanging performance (8.83 ± 1.80 vs 116.33 ± 3.67 sec), along with increased immobility-related and anxiety-like behavioral changes (P < 0.001). Treatment with captopril, perindopril, and losartan significantly improved motor performance and behavioral outcomes, with locomotor activity ranging from 141.83 to 154.50 counts/10 min and rotarod performance from 108.67 to 114.17 sec. Significant reductions in immobility time and improvements in exploratory and anxiety-related parameters were also observed (P < 0.001).

Conclusion: RAS modulation using angiotensin-converting enzyme inhibitors and angiotensin receptor blockers demonstrated significant improvement in motor and behavioral parameters in a rotenone-induced PD model. These findings suggest the potential therapeutic utility of these agents in mitigating both motor and non-motor manifestations associated with PD. However, further biochemical, histopathological, mechanistic, and clinical investigations are required to establish their potential neuroprotective role.

## Introduction

Parkinson’s disease (PD) is a progressive neurodegenerative disorder characterized primarily by motor dysfunction, including bradykinesia, rigidity, resting tremor, and postural instability, resulting from the degeneration of dopaminergic neurons in the substantia nigra pars compacta [1]. In addition to motor symptoms, patients frequently exhibit non-motor manifestations such as depression, anxiety, and cognitive impairment, which significantly contribute to disease burden and reduced quality of life [2]. The pathophysiology of PD is complex and involves multiple mechanisms, including oxidative stress, mitochondrial dysfunction, neuroinflammation, and protein aggregation [3].

Experimental models play a crucial role in understanding the pathogenesis of PD and evaluating potential therapeutic interventions [4]. Among these, the rotenone-induced model is widely used because it closely mimics the pathological and behavioral features of PD [5]. Rotenone, a mitochondrial complex I inhibitor, induces dopaminergic neuronal degeneration through enhanced oxidative stress and impaired energy metabolism, thereby reproducing both motor deficits and non-motor behavioral alterations observed in PD [6]. This model has been extensively utilized for screening potential therapeutic agents and studying disease-related behavioral alterations.

The renin-angiotensin system (RAS), traditionally associated with cardiovascular regulation, has emerged as an important modulator within the central nervous system [7]. Increasing evidence suggests that dysregulation of brain RAS contributes to neurodegeneration through mechanisms such as oxidative stress, inflammation, and apoptosis [8]. Pharmacological agents targeting RAS, including angiotensin-converting enzyme (ACE) inhibitors such as captopril and perindopril, and angiotensin receptor blockers (ARBs) such as losartan, have demonstrated beneficial effects on neuronal function and behavioral outcomes in various experimental models and have been proposed to possess potential neuroprotective properties [9,10].

Given the involvement of RAS in neurodegenerative pathways and the need for effective disease-modifying therapies in PD, evaluating the neurobehavioral effects of these agents in validated experimental models is of considerable importance. Therefore, the present study aimed to assess the effects of captopril, perindopril, and losartan on motor function, exploratory behavior, immobility-related behavioral changes, and anxiety parameters in a rotenone-induced model of PD in Wistar albino rats.

## Materials and methods

The present experimental in vivo study was conducted following approval from the Institutional Animal Ethics Committee, Shri B. M. Patil Medical College, BLDE (Deemed to be University), Vijayapura (IEC No: 33/16), registered under the Committee for the Purpose of Control and Supervision of Experiments on Animals (CPCSEA), India. All experimental procedures were performed in accordance with CPCSEA guidelines for the care and use of laboratory animals. A minimum sample size of six animals per group was considered adequate to obtain statistically valid results, as per standard experimental protocols [11,12].

Adult male Wistar albino rats were used for the rotenone model due to their ease of handling, reproducibility, and physiological similarity to humans [13]. Animals were maintained under standard laboratory conditions with controlled temperature, humidity, and a 12-hour light-dark cycle, with free access to standard pellet diet and water. Animals were randomly allocated into six experimental groups using a simple randomization method, and the treatment protocol is summarized in Table 1.

**Table 1:** Experimental groups in rotenone model. i.p., intraperitoneal; BW, body weight.

Group	Description	Number of rats (n)
Group I	Vehicle control: Normal saline (i.p.)	6
Group II	Rotenone (3 mg/kg BW i.p. daily for 21 days) – Negative control	6
Group III	Levodopa (12 mg/kg BW) + Benserazide (3 mg/kg BW i.p. daily for 25 days) + Rotenone (3 mg/kg BW i.p. daily for 21 days) – Standard Treatment group	6
Group IV	Captopril (20 mg/kg BW i.p. daily for 25 days) + Rotenone (3 mg/kg BW i.p. daily for 21 days)	6
Group V	Perindopril (5 mg/kg BW i.p. daily for 25 days) + Rotenone (3 mg/kg BW i.p. daily for 21 days)	6
Group VI	Losartan (90 mg/kg BW i.p. daily for 25 days) + Rotenone (3 mg/kg BW i.p. daily for 21 days)	6

Rotenone solution was freshly prepared at a dose of 3 mg/kg by dissolving it in dimethyl sulfoxide (DMSO) and adjusting the pH to 7.4 using potassium hydroxide prior to administration. The solution was used immediately after preparation due to its limited stability at room temperature. All drugs were administered intraperitoneally as per the respective dosing schedules. Rotenone (Sigma-Aldrich, St. Louis, Missouri, USA), levodopa and benserazide (Sun Pharmaceutical Industries Ltd, Mumbai, Maharashtra, India), captopril (Cipla Ltd, Mumbai, Maharashtra, India), perindopril (Servier India Pvt. Ltd, New Delhi, India), losartan (Torrent Pharmaceuticals Ltd, Ahmedabad, Gujarat, India), dimethyl sulfoxide (Merck Life Science Pvt. Ltd, Mumbai, Maharashtra, India), and potassium hydroxide (Loba Chemie Pvt. Ltd, Mumbai, Maharashtra, India) were used in the present study.

Behavioral assessments and statistical analysis were performed by investigators blinded to treatment allocation to minimize observer bias. All behavioral assessments were conducted during the light phase between 9:00 AM and 1:00 PM to minimize circadian variability.

Neurobehavioral assessment was carried out using validated experimental paradigms to evaluate motor function, exploratory behavior, immobility-related behavioral changes, and anxiety. Spontaneous locomotor activity was assessed using an actophotometer based on photoelectric beam interruptions and expressed as counts per 10 minutes [14]. Motor coordination was evaluated using the rotarod apparatus, where latency to fall (maximum cut-off 240 seconds) was recorded [15]. Animals were trained on the rotarod apparatus prior to experimentation to ensure familiarization with the procedure. Neuromuscular performance was assessed using a wire-hanging test, in which the duration for which the animal could maintain grip on a suspended thread was recorded [16]. Exploratory behavior was evaluated using the hole board test, where the number of head dips over a five-minute period was recorded [17]. Behavioral immobility was assessed using the forced swim test and tail suspension test; however, these findings were interpreted cautiously because severe motor impairment in rotenone-treated animals may confound immobility-based behavioral outcomes [18,19]. Anxiety-related behavior was evaluated using the elevated plus maze, where parameters such as percentage open arm preference, calculated as (number of open arm entries ÷ total arm entries) × 100, number of entries made into open and closed arms, and time spent in open arms were recorded over a five-minute period [20].

Statistical analysis was performed using IBM SPSS Statistics for Windows, Version 26.0 (IBM Corp., Armonk, NY, USA) using one-way analysis of variance (ANOVA) followed by Tukey’s post hoc test for multiple comparisons. Data were expressed as mean ± standard error (SE), and a value of P < 0.05 was considered statistically significant.

## Results

Rotenone administration in the disease control group (Group II) caused a marked decline in motor and neuromuscular function, with locomotor activity reduced to 18.67 ± 4.11 counts/10 min, rotarod performance to 10.17 ± 1.19 sec, and wire-hanging performance to 8.83 ± 1.80 sec, compared to the vehicle control group (Group I: 174.33 ± 25.01 counts/10 min, 118.17 ± 1.83 sec, and 116.33 ± 3.67 sec, respectively). Treatment with the standard treatment group (Group III) significantly restored these parameters (161.33 ± 15.25 counts/10 min, 116.17 ± 2.59 sec, and 113.17 ± 6.83 sec). All experimental groups (IV-VI) showed significant improvement, with locomotor activity ranging from 141.83 to 154.50 counts/10 min, rotarod performance from 108.67 to 114.17 sec, and wire-hanging performance from 106.83 to 111.50 sec, approaching normal values. Overall differences across groups were statistically significant for all parameters (P < 0.001) (Tables 2, 3).

**Table 2:** Motor and neuromuscular performance. Data are presented as mean ± SE. Statistical analysis was performed using one-way ANOVA. BW, body weight; sec, seconds; min, minutes. A p-value <0.05 was considered statistically significant.

Parameter	Group I (n=6)	Group II (n=6)	Group III (n=6)	Group IV (n=6)	Group V (n=6)	Group VI (n=6)	F-value	P-value
Locomotor activity (counts/10 min) Mean ± SE	174.33 ± 25.01	18.67 ± 4.11	161.33 ± 15.25	149.83 ± 17.04	141.83 ± 9.16	154.50 ± 15.42	32.41	<0.001
Rotarod (sec) Mean ± SE	118.17 ± 1.83	10.17 ± 1.19	116.17 ± 2.59	110.17 ± 7.01	108.67 ± 6.49	114.17 ± 5.83	45.62	<0.001
Wire-hanging test (sec) Mean ± SE	116.33 ± 3.67	8.83 ± 1.80	113.17 ± 6.83	109.17 ± 6.88	106.83 ± 8.34	111.50 ± 8.50	38.75	<0.001

**Table 3:** Post hoc Tukey’s test for motor and neuromuscular performance.

Comparison	Locomotor activity p-value	Rotarod p-value	Wire-hanging test p-value
Group I vs. Group II	<0.001	<0.001	<0.001
Group I vs. Group III	0.842	0.913	0.887
Group I vs. Group IV	0.654	0.731	0.702
Group I vs. Group V	0.518	0.624	0.587
Group I vs. Group VI	0.773	0.851	0.826
Group II vs. Group III	<0.001	<0.001	<0.001
Group II vs. Group IV	<0.001	<0.001	<0.001
Group II vs. Group V	<0.001	<0.001	<0.001
Group II vs. Group VI	<0.001	<0.001	<0.001
Group III vs. Group IV	0.794	0.688	0.742
Group III vs. Group V	0.563	0.591	0.527
Group III vs. Group VI	0.911	0.936	0.902
Group IV vs. Group V	0.837	0.873	0.846
Group IV vs. Group VI	0.902	0.921	0.915
Group V vs. Group VI	0.741	0.782	0.754

The disease control group (Group II) exhibited significant behavioral impairment, with reduced exploratory activity (6.33 ± 1.26 nose pokings) and increased immobility in tail suspension (179.17 ± 20.26 sec) and forced swim tests (165.33 ± 9.12 sec) compared to the vehicle control (35.50 ± 3.63, 26.50 ± 4.30, and 31.33 ± 3.64, respectively). The standard treatment group (Group III) significantly reversed these effects (31.17 ± 3.46, 29.67 ± 4.71, and 34.50 ± 3.00). Experimental groups (IV-VI) also demonstrated notable improvement, with hole board activity ranging from 25.33 to 29.50, and immobility times reduced to 32.33-41.17 sec (tail suspension) and 38.33-44.33 sec (forced swim). All parameters showed statistically significant differences across groups (F = 27.83, 52.14, and 48.67; P < 0.001) (Tables 4, 5). However, immobility-related findings were interpreted cautiously because severe motor impairment induced by rotenone may contribute to increased immobility independent of depressive-like behavior.

**Table 4:** Behavioral and immobility-related parameters. Data are presented as mean ± SE. Statistical analysis was performed using one-way ANOVA. sec, seconds; no., number. A p-value <0.05 was considered statistically significant.

Parameter	Group I (n=6)	Group II (n=6)	Group III (n=6)	Group IV (n=6)	Group V (n=6)	Group VI (n=6)	F-value	P-value
Hole board (no. of nose pokings) Mean ± SE	35.50 ± 3.63	6.33 ± 1.26	31.17 ± 3.46	28.50 ± 2.29	25.33 ± 3.28	29.50 ± 3.70	27.83	<0.001
Tail suspension test (sec) Mean ± SE	26.50 ± 4.30	179.17 ± 20.26	29.67 ± 4.71	37.17 ± 3.11	41.17 ± 3.28	32.33 ± 2.89	52.14	<0.001
Forced swim test (sec) Mean ± SE	31.33 ± 3.64	165.33 ± 9.12	34.50 ± 3.00	41.50 ± 1.91	44.33 ± 2.94	38.33 ± 3.39	48.67	<0.001

**Table 5:** Post hoc Tukey’s test for behavioral and immobility-related parameters.

Comparison	Hole board p-value	Tail suspension test p-value	Forced swim test p-value
Group I vs. Group II	<0.001	<0.001	<0.001
Group I vs. Group III	0.804	0.882	0.867
Group I vs. Group IV	0.641	0.706	0.693
Group I vs. Group V	0.532	0.618	0.584
Group I vs. Group VI	0.776	0.853	0.821
Group II vs. Group III	<0.001	<0.001	<0.001
Group II vs. Group IV	<0.001	<0.001	<0.001
Group II vs. Group V	<0.001	<0.001	<0.001
Group II vs. Group VI	<0.001	<0.001	<0.001
Group III vs. Group IV	0.818	0.754	0.737
Group III vs. Group V	0.594	0.563	0.541
Group III vs Group VI	0.926	0.908	0.897
Group IV vs. Group V	0.853	0.881	0.862
Group IV vs. Group VI	0.918	0.934	0.916
Group V vs. Group VI	0.766	0.791	0.772

Rotenone significantly induced anxiety-like behavior in the disease control group (Group II), as indicated by reduced open arm preference (16.67 ± 4.08%), decreased open arm entries (3.17 ± 0.48), increased closed arm entries (17.83 ± 1.51), and reduced time spent in open arms (17.50 ± 2.86 sec) compared to the vehicle control group (83.33 ± 5.16%, 16.50 ± 1.12, 2.67 ± 0.71, and 168.67 ± 10.73 sec, respectively). Treatment with the standard treatment group (Group III) markedly improved these parameters (83.33 ± 4.08%, 14.67 ± 1.58, 3.17 ± 0.87, and 156.83 ± 11.22 sec). Experimental groups (IV-VI) also showed significant anxiolytic effects, with open arm preference ranging from 50.00 ± 6.32% to 66.67 ± 5.16%, open arm entries ranging from 10.50 to 12.67, closed arm entries from 3.67 to 4.50, and time spent in open arms from 140.67 to 145.17 sec. All anxiety-related parameters differed significantly across experimental groups (P < 0.001) (Tables 6, 7).

**Table 6:** Elevated plus maze (anxiety parameters). Data are presented as mean ± SE. Statistical analysis was performed using one-way ANOVA. sec, seconds; %, percentage. A p-value <0.05 was considered statistically significant. % open arm preference was calculated as: (number of open arm entries ÷ total arm entries) × 100. Open arm entries and closed arm entries represent the number of entries made into the respective arms during the five-minute observation period.

Parameter	Group I (n=6)	Group II (n=6)	Group III (n=6)	Group IV (n=6)	Group V (n=6)	Group VI (n=6)	F-value	P-value
% open arm preference Mean ± SE	83.33 ± 5.16	16.67 ± 4.08	83.33 ± 4.08	66.67 ± 5.16	50.00 ± 6.32	66.67 ± 5.16	21.45	<0.001
Open arm entries Mean ± SE	16.50 ± 1.12	3.17 ± 0.48	14.67 ± 1.58	11.67 ± 1.05	10.50 ± 1.23	12.67 ± 1.20	33.76	<0.001
Closed arm entries Mean ± SE	2.67 ± 0.71	17.83 ± 1.51	3.17 ± 0.87	4.17 ± 0.65	4.50 ± 0.76	3.67 ± 0.49	41.22	<0.001
Time in open arm (sec) Mean ± SE	168.67 ± 10.73	17.50 ± 2.86	156.83 ± 11.22	142.83 ± 6.73	140.67 ± 5.52	145.17 ± 9.71	36.18	<0.001

**Table 7:** Post hoc Tukey’s test for elevated plus maze parameters.

Comparison	% open arm preference p-value	Open arm entries p-value	Closed arm entries p-value	Time in open arm p-value
Group I vs. Group II	<0.001	<0.001	<0.001	<0.001
Group I vs. Group III	0.998	0.874	0.893	0.861
Group I vs. Group IV	0.624	0.703	0.721	0.694
Group I vs. Group V	0.482	0.587	0.601	0.573
Group I vs. Group VI	0.651	0.742	0.758	0.736
Group II vs. Group III	<0.001	<0.001	<0.001	<0.001
Group II vs. Group IV	<0.001	<0.001	<0.001	<0.001
Group II vs. Group V	<0.001	<0.001	<0.001	<0.001
Group II vs. Group VI	<0.001	<0.001	<0.001	<0.001
Group III vs. Group IV	0.553	0.641	0.658	0.629
Group III vs. Group V	0.417	0.526	0.544	0.512
Group III vs. Group VI	0.586	0.673	0.688	0.661
Group IV vs. Group V	0.792	0.851	0.864	0.843
Group IV vs. Group VI	0.911	0.924	0.936	0.918
Group V vs. Group VI	0.754	0.803	0.817	0.788

## Discussion

The present study demonstrated that rotenone administration produced profound motor deficits, as evidenced by a marked reduction in locomotor activity, rotarod performance, and wire-hanging performance, consistent with nigrostriatal dopaminergic dysfunction. These findings align with earlier experimental reports showing that rotenone, through inhibition of mitochondrial complex I, induces oxidative stress and selective dopaminergic neuronal degeneration, thereby reproducing key motor features of Parkinson’s disease. Studies by Sherer et al. and Betarbet et al. have similarly demonstrated significant impairment in motor coordination and locomotor activity in rotenone-treated rodents, supporting the behavioral validity of the present model [21,22]. The observed restoration of motor function with levodopa and benserazide further supports the dopaminergic basis of these deficits, while the significant improvement seen with captopril, perindopril, and losartan suggests a potential beneficial effect of renin-angiotensin system (RAS) modulators on rotenone-induced behavioral impairment. The significant improvement in locomotor activity, rotarod performance, and wire-hanging performance observed in the treatment groups indicates that RAS modulation may mitigate rotenone-induced motor dysfunction. However, no biochemical or histopathological analyses were performed to directly confirm neuroprotection or preservation of dopaminergic neurons.

In addition to motor impairment, rotenone induced significant behavioral alterations indicative of reduced exploratory activity and increased immobility-related behavioral changes, as evidenced by decreased hole board activity and increased immobility in tail suspension and forced swim tests. These findings are consistent with previous studies reporting non-motor manifestations in rotenone models, including reduced motivation and altered behavioral responses. Experimental evidence by Bové & Perier highlights the role of mitochondrial dysfunction and oxidative stress in mediating such behavioral changes [23]. In the present study, treatment with ACE inhibitors and the ARB significantly reduced immobility time and improved exploratory behavior. Similar neurobehavioral improvements have been reported with RAS-modulating agents and may possibly be related to previously described antioxidant and anti-inflammatory effects of these agents. However, interpretation of immobility-based behavioral tests should be made cautiously, as severe motor impairment induced by rotenone may contribute to increased immobility independent of depressive-like behavior. The observed reduction in immobility time and restoration of exploratory behavior therefore suggest an improvement in behavioral performance in rotenone-treated animals.

Anxiety-like behavior assessed using the elevated plus maze revealed that rotenone significantly reduced open arm preference, open arm entries, and time spent in open arms, while increasing closed arm entries, indicating heightened anxiety-related behavior. These observations are in agreement with established literature demonstrating that neurodegeneration in PD models is associated with altered limbic system function and anxiety-related behavioral changes. The significant reversal of these parameters by losartan, captopril, and perindopril in the present study is consistent with previous findings suggesting anxiolytic-like effects of RAS inhibitors. Studies by Wright & Harding have demonstrated that central angiotensin II plays a role in stress and anxiety modulation, and blockade of this pathway can improve behavioral outcomes [24]. The improvement in elevated plus maze performance observed in the treatment groups suggests that modulation of central RAS pathways may contribute to attenuation of anxiety-related behavior in Parkinsonian conditions.

The neurobehavioral improvements observed with captopril, perindopril, and losartan in the present study suggest a potential role of renin-angiotensin system modulation in attenuating rotenone-induced behavioral abnormalities. These effects may possibly be associated with antioxidant and anti-inflammatory mechanisms previously described in experimental studies. Previous investigations have similarly demonstrated that inhibition of angiotensin signalling can attenuate oxidative stress and neuroinflammation, thereby potentially contributing to improved neuronal function. The observed benefits across motor, behavioral, and anxiety parameters in the present study further support the possible involvement of central RAS pathways in neurodegenerative processes. However, the exact molecular mechanisms underlying these effects remain unclear and require further exploration through biochemical and histopathological investigations. Future studies incorporating neurotransmitter analysis, inflammatory markers, oxidative stress parameters, and histopathological assessment may provide greater insight into the mechanistic basis of the observed behavioral improvements.

Strengths and limitations

The present study has several strengths, including the use of a well-established rotenone-induced model that closely mimics both motor and non-motor features of Parkinson’s disease, along with a comprehensive neurobehavioral assessment covering locomotor, exploratory, immobility-related, and anxiety parameters. The inclusion of both ACE inhibitors and an angiotensin receptor blocker allowed for a comparative evaluation of different components of the renin-angiotensin system, enhancing the translational relevance of the findings.

However, certain limitations should be acknowledged. The study relied primarily on behavioral outcomes without incorporating biochemical, molecular, neurotransmitter, or histopathological analyses to elucidate underlying mechanisms or confirm dopaminergic neuronal preservation. The relatively small sample size (n = 6 per group), though standard for preclinical studies, may limit generalizability, and a formal power calculation was not performed. Additionally, the short duration of assessment and absence of dose-response evaluation restrict the understanding of long-term and optimal therapeutic effects. The use of tail suspension testing in rats and the potential confounding influence of severe motor impairment on immobility-based behavioral tests should also be considered while interpreting the findings. Furthermore, a matched vehicle control for the rotenone preparation was not included, which may represent an additional methodological limitation. Although the concentration of DMSO used for rotenone preparation was minimal, the absence of a dedicated DMSO vehicle control should be considered while interpreting the findings.

Despite these limitations, the present findings provide preliminary behavioral evidence supporting the potential beneficial effects of RAS modulators in rotenone-induced Parkinsonian models and justify further translational and mechanistic investigations.

## Conclusions

The present study demonstrates that modulation of the renin-angiotensin system using captopril, perindopril, and losartan significantly attenuates motor deficits, immobility-related behavioral changes, and anxiety-related behavior in a rotenone-induced model of Parkinson’s disease. These findings suggest that both ACE inhibitors and angiotensin receptor blockers exert beneficial effects on behavioral outcomes in rotenone-treated animals. The observed improvements across multiple neurobehavioral domains highlight the potential behavioral benefits of these agents as adjunctive therapeutic strategies in Parkinson’s disease. The findings also support the emerging role of central renin-angiotensin system pathways in the pathogenesis and progression of neurodegenerative disorders.

However, the present study did not include biochemical, molecular, neurotransmitter, or histopathological analyses to directly confirm neuroprotection or elucidate underlying mechanisms. Therefore, the observed effects should be interpreted as preliminary behavioral findings rather than definitive evidence of neuroprotection. Further studies incorporating detailed mechanistic, biochemical, histopathological, and clinical evaluations are required to substantiate these findings and facilitate their translation into clinical practice. Long-term studies with larger sample sizes and dose-response analyses are additionally warranted to establish the therapeutic applicability and safety profile of these agents in Parkinson’s disease.
